# The best treatment is prevention: prevention of cognitive decline and dementia – current state, gaps and next steps –

**DOI:** 10.1186/s42466-026-00494-4

**Published:** 2026-04-21

**Authors:** Kathrin Reetz, Inga Liepelt-Scarfone, Alexa Häger, Agnes Flöel, Jörg B. Schulz

**Affiliations:** 1 Department of Neurology, Centre for Dementia and Prevention Aachen (ZDPA), RTWH Aachen University, Aachen, Germany; 2 Hertie Institute for Clinical Brain Research and German Center for Neurodegenerative Diseases (DZNE), University of Tubingen, Tubingen, Germany; 3 IB Hochschule für Gesundheit und Soziales, Stuttgart, Germany; 4 Department of Neurology, University Medicine, 17475 Greifswald, Germany; 5 German Center for Neurodegenerative Diseases (DZNE), Standort Rostock/Greifswald, Greifswald, Germany; 6 Department of Neurology, Pauwelsstraße 30, 52074 Aachen, Germany

**Keywords:** Prevention, Alzheimer’s disease, Cognitive decline, Dementia, Risk factors, Treatment, Public health, Education

## Abstract

The World Health Organization (WHO) recognises dementia as a public health priority. Currently, more than 55 million people live with dementia, and this figure is expected to almost triple to 139 million by 2050. Alzheimer’s disease is the most common cause of dementia, and there is still no cure. Despite the availability of symptomatic and first disease-modifying treatments, effective treatment remains limited. This makes it even more important to be aware that it is estimated that nearly half of all dementia cases could be prevented by eliminating 14 major risk factors. Consequently, the role of these modifiable risk factors has been the subject of research to inform effective preventive strategies. This narrative review summarises the current evidence on the prevention of cognitive decline and dementia. First, we provide an overview of the *current state* on risk factors, multimodal clinical controlled trials for slowing down or preventing dementia and primordial, primary, secondary, tertiary and quaternary prevention of cognitive decline and dementia. Second, we evaluate knowledge and action *gaps* in the field. Finally, we discuss how to address these gaps and what the *next steps* should be. Overall, to accelerate progress, the following key calls to action are paramount: (1) making prevention of cognitive decline and dementia a priority; (2) promoting brain health throughout the lifespan through coordinated intersectoral action targeting key risk factors; (3) improving access to diagnosis and treatment for patients with cognitive decline and dementia; and (4) encouraging research on prevention and how to translate knowledge into action.

## Introduction

The World Health Organization (WHO) recognizes dementia as a public health priority. According to the WHO, more than 55 million people currently live with dementia, and this number is expected to nearly triple to 139 million by 2050 [1]. Dementia is currently the seventh leading cause of death worldwide and the fifth leading cause among noncommunicable diseases [2].

“Brain health” is the state of brain function in the areas of cognition, sensory perception, socio-emotionality, behaviour, and motor skills that enables a person to reach their full potential throughout their life, regardless of the presence or absence of disorders; as defined by the WHO [3]. Maintaining brain health usually involves a healthy lifestyle, engaging in activities that stimulate the brain, and taking measures to prevent neurological diseases and their devastating symptoms, such as cognitive decline and dementia.

Alzheimer’s disease (AD) is the most common cause of dementia, accounting for 60–70% of cases [4]. Although new treatments are underway, there is currently no cure for dementia. It can be assumed that several different therapeutic approaches will probably be necessary for an effective treatment, with the most promising and socioeconomically favourable (cost-benefit analysis with relief for health insurance companies) intervention being prevention – “the best treatment is prevention” [5, 6].

There have been several initiatives to address this public health priority. In 2017, the World Health Assembly endorsed the Global Action Plan on the Public Health Response to Dementia 2017–2025 [7]. The global action plan provides a comprehensive blueprint for action – for policy-makers, international, regional and national partners, and WHO in the following areas: addressing dementia as a public health priority; increasing awareness of dementia and creating a dementia-inclusive society; reducing the risk of dementia; diagnosis, treatment and care; information systems for dementia; support for carer for patients with dementia; and research and innovation.

At the European level, the European Academy of Neurology (EAN) has recently (2025) provided “a roadmap toward promoting and improving brain health in Europe and closing the awareness and funding gap” [8]. This initiative aims to enhance lifelong brain health, reduce the prevalence and impact of related diseases, as investing in brain health can lower healthcare costs, yield long-term economic benefits, and significantly improve well-being [8].

At the national level, three major actions in Germany need to be emphasized. First, in 2022, the German Brain Council presented the ‘German Brain Plan – Agenda 2030’, proposing that the maintenance of stable human health is contingent upon the preservation of brain health as based on the fact that 25 per cent of the global population is currently affected by a neurological or mental illness, which results in annual costs of more than €800 billion in Europe alone [9]. Second, the recent national guidelines for dementia recommend that healthcare professionals should consider modifiable risk factors when counselling patients with mild cognitive impairment and dementia [10]. Third, with this paper on the prevention of cognitive decline and dementia, and within other neurological indications, the German Neurological Society (DGN) focuses on the prevention of neurological diseases at every stage of life with strategies that advance awareness, education, individualized treatment and public health. Thus, herewith we are aiming to draw attention to this public health priority and provide current research, knowledge and potential strategies on the prevention of cognitive decline and dementia.

## Current state on risk factors

In 2020, it has been estimated that modifying the following risk factors across the lifespan could prevent around 40% of dementia cases [11]: lower educational status in early life; diabetes; hypertension; hearing loss; physical inactivity; excessive alcohol consumption; smoking; obesity; traumatic brain injury; depression in middle life; and social isolation, and air pollution in later life. In 2024, an updated review has verified the influence of the afore mentioned markers on the conversion to dementia and identified the two additional factors, i.e. middle-age high LDL-cholesterol and vision loss [6], resulting in an estimated reduction of 45% [6] of dementia cases by applying appropriate prevention strategies to these persons at risk. It is well known that optimising and treating those modifiable risk factors can diminish vascular damage, stress and inflammatory processes, strengthen the brain’s mental functioning and performance (cognitive reserve), and reduce underlying disease mechanisms. Worldwide, especially the treatment of hearing loss and high LDL cholesterol in middle age, and the avoidance of social isolation in later life, appear seem to have a significant impact on dementia prevention [6]. However, it should be noted that treatment recommendations may differ between countries. For example, the most relevant current risk factors estimate for Germany are hearing loss, arterial hypertension, depression, obesity and smoking [5]. Besides these single factors, especially individuals with physical and mental multimorbidity are at a higher risk of dementia [12], which highlights the importance of early multidisciplinary identification and treatment.

Although many studies have focused on identifying potential risk factors, it is currently unclear whether dementia risk should be personalised or stratified according to a single risk profile, probably due to the fact that the false positive rate of each individual marker is high. Here, a composite modifiable risk score, such as the LIfestyle for BRAin Health (LIBRA) index [13] or the Australian National University Alzheimer’s Disease Risk Index (ANU-ADRI) [14] have the advantage to reflect the syndrome’s multifactorial etiology. First results have demonstrated that an increase of one-standard-deviation in the LIBRA index is associated with a 21% higher risk of dementia [13]. This supports the idea that a composite risk score reflecting brain health is relevant for reducing dementia risk across diverse geographical and sociodemographic groups [13]. However, further research is required to assess the effectiveness of these composite scores in preventing dementia in a clinical care setting.

The current national S3 guideline for dementia in Germany [10] already recommends taking potentially modifiable risk factors into account during consultations. Unfortunately, the healthcare system still does not adequately acknowledge the importance of maintaining brain health and preventing dementia. To close this gap in care, a multidisciplinary team approach is required. Close cooperation with family doctors and general practitioners is also necessary to further reduce the risk of dementia in the future.

### Current state on multimodal clinical controlled trials for slowing down or preventing dementia in the context of disease stages

Identifying effective strategies to slow or prevent cognitive decline in Alzheimer’s and other dementias is a key public health priority, driven by rising prevalence and associated economic, psychological, and social burdens. Since age-related decline often results from mixed pathology [15], successful treatment will likely require a multimodal approach addressing multiple mechanisms, including Alzheimer’s and vascular pathology. Most multidomain interventions primarily aim to strengthen cognitive reserve. These approaches enhance the brain’s ability to maintain function despite underlying neuropathological changes. While they do not halt the accumulation of β-amyloid or tau in Alzheimer’s disease, they can improve resilience and delay the clinical expression of cognitive decline.

Various randomized-controlled trials (RCTs) have investigated strategies for dementia prevention. While early clinical trials mostly focused on individual risk factors, large-scale multimodal randomised controlled trials are now investigating the effectiveness of complex lifestyle interventions that simultaneously address multiple modifiable risk factors in participants with an increased risk of dementia or in preclinical/prodromal stages (Table 1).

**Table 1:** Multimodal clinical controlled trials for slowing down or preventing dementia

Name and reference of the intervention study	Intervention	Study design	Main result
**FINGER** [16] **(2015)** Finnish Geriatric Intervention Study to Prevent Cognitive Impairment and Disability (FINGER)	Multidomain intervention (nutritional counselling, physical activity, cognitive training, management of cardiovascular risk factors) vs. control group (standard health counselling)	*Design*: Randomised controlled trial *Duration*: 2 years *Study participants*: 1,260 older adults (aged 60–77) with an increased risk of dementia *Location*: Finland	The multidomain intervention improved global cognitive function. The estimated mean change in the NTB total z-score after 2 years was 0.20 (SE 0.02, SD 0.51) in the intervention group and 0.16 (0.01, 0.51) in the control group. The group difference in the change in the NTB total score per year was 0.022 (95% CI 0.002 to 0.042, *P* = 0.030).
**PreDIVA** [19] **(2016)** 6-year multidomain vascular care intervention to prevent dementia (preDIVA): a cluster-randomised controlled trial ISRCTN29711771	Multidomain cardiovascular intervention led by nurses or control (usual care)	*Design*: Cluster-randomised controlled trial *Duration*: 6 years (2006–2009) *Study participants*: 3,526 older adults (aged 70–78) *Location*: *N* = 116 general practices within 26 health sites in the Netherlands	Dementia developed in 121 (7%) in the intervention group and in 112 (7%) in the control group (HR 0.92, 95% CI 0.71–1.19, *P* = 0.54). The mean dementia and disability score (Academic Medical Centre Linear Disability Score [ALDS]) did not differ between the two groups
**MAPT **[17] **(2017)** Long-term omega 3 polyunsaturated fatty acid supplementation with or without multidomain intervention on cognitive function in elderly adults with memory complaints (MAPT) NCT00672685^#^	Multidomain intervention on the effect of supplementation with polyunsaturated omega-3 fatty acids and a multi-stage intervention (physical activity, cognitive training and nutritional counselling), alone or in combination, compared to placebo, on cognitive decline	*Design*: Multicentre, randomised, placebo-controlled study with 4 parallel groups *Duration*: 3 years (2008–2011) *Study participants*: 1,680 older adults (aged 70 or older) *Location*: 13 memory clinics in France and Monaco	No significant differences in cognitive decline after 3 years between any of the three intervention groups and the placebo group. The differences between the groups compared to placebo were 0.093 (95% CI 0.001 to 0.184; adjusted *p* = 0.142) for the combined intervention group, 0.079 (−0.012 to 0.170; 0.179) for the multidimensional intervention plus placebo group, and 0.011 (−0.081 to 0.103; 0.812) for the polyunsaturated omega-3 fatty acid group
**Look AHEAD **[27] **(2017)** *Action for Health in Diabetes* Long-term intensive lifestyle intervention on prevalence of cognitive impairment NCT00017953^#^	Multidomain lifestyle intervention (diabetes support and counselling, physical activity)	*Design*: Randomised controlled trial *Duration*: 10 years *Study participants*: 3,802 older adults (aged 45–76 with type II diabetes and overweight (BMI > 25 kg/m^2^) *Location*: 18 sites in the USA	The lifestyle intervention resulted in weight loss and maintenance compared to the control group. The prevalence of MCI and dementia was 6.4% and 1.8% in the intervention group versus 6.6% and 1.8% in the control group (*P* = 0.93)
**HATICE **[18] **(2019)** Healthy ageing through internet counselling in the elderly (HATICE) ISRCTN48151589^#^	Interactive internet intervention with support from a coach to promote self-management (potential health priorities: smoking, blood pressure, cholesterol, diabetes, weight, physical activity and nutrition)	*Design*: Randomised controlled trial *Duration*: 18 months *Study participants*: 2,724 older adults (aged 65 or older) *Location*: sites in France, Finland and the Netherlands	The primary outcome improved in the intervention group compared to the control group (0.09 vs. 0.04; mean difference − 0.05, 95% CI −0.08 to −0.01; *p* = 0.008): Intervention vs. control: systolic blood pressure − 1.79 mm Hg vs. −0.67 mm Hg; BMI − 0.23 kg/m² vs. −0.08 kg/m²; LDL − 0.12 mmol/L vs. −0.07 mmol/L. Cardiovascular events occurred in 30 (2–2%) of 1,382 patients in the intervention group compared with 32 (2–4%) of 1,333 patients in the control group (hazard ratio 0.86, 95% CI 0.52 to 1.43)
**BBL-CD **[20] **(2020)** Lifestyle Risk Factors and Cognitive Outcomes from the Multidomain Dementia Risk Reduction Randomised Controlled Trial, Body Brain Life for Cognitive Decline (BBL-CD)	Multidomain lifestyle intervention (online educational modules and additional active components: sessions with nutritionists, one session with exercise physiotherapists and brain training)	*Design*: Randomised controlled trial *Duration*: 8 weeks *Study participants*: 119 older adults with SCI or MCI *Location*: Australia	The intervention group showed a lower ANU-ADRI score (χ2 = 10.84; df = 3; *P* = 0.013) and a higher cognition score (χ2 = 7.28; df = 2; *P* = 0.026) than the control group. A secondary analysis showed that lifestyle changes were attributable to an increase in protective lifestyle factors (χ2 = 12.02; df = 3; *P* = 0.007) rather than a reduction in risk factors (χ2 = 2.93; df = 3; *P* = 0.403), and cognitive changes were only evident for the SDMT (χ2 = 6.46; df = 2; *P* = 0.040)
**SMARRT study **[23] **(2024)** NCT03683394^#^	Personalised risk reduction goals with health counselling and nurse visits or a control group for health education	*Design*: Multidomain randomised clinical intervention trial *Duration*: 2 years *Study participants*: 172 older adults (aged 70–89) *Location*: 5 sites in Germany	After two years, the 82 participants who took part in the intervention showed greater improvements in composite cognitive score (ATE of SD, 0.14; 95% CI, 0.03–0.25; *P* = 0.02; a 74% improvement compared to the change in the control group), a better composite risk factor score (ATE of SD, 0.11; 95% CI, 0.01–0.20; *P* = 0.03) and improved quality of life (ATE, 0.81 points; 95% CI, −0.21 to 1.84; *P* = 0.12)
**AGE-Well **[24] **(2024)** DRKS00013555^#^	Multidomain intervention (optimisation of nutrition, medication, and physical, social, and cognitive activity)	*Design*: Multidomain intervention study *Duration*: 2 years *Study participants*: 461 older adults *Location*: 5 sites in Germany	The intervention reduced LIBRA scores, suggesting a lower risk of dementia at follow-up (b = −0.63, 95% confidence interval [CI]: −1.14, −0.12). The intervention effects were particularly attributable to improvements in nutrition (odds ratio [OR]: 1.60, 95% CI: 1.16, 2.22) and high blood pressure (OR: 1.61, 95% CI: 1.19, 2.18)
**POINTER **[25] **(2025)** Structured vs. Self-Guided Multidomain Lifestyle Interventions for Global Cognitive Function; The US POINTER Randomised Clinical Trial NCT03688126^#^	Structured or self-guided intervention involving physical and cognitive activity, healthy eating, social engagement and cardiovascular health	*Design*: multicentre, randomised clinical trial *Duration*: 2-year period *Study participants*: 2,111 aged 60–79 (1,056 structured group; 1,055 self-directed group) *Location*: 5 sites in the USA	Mean global cognitive composite Z-score increased over time from baseline in both groups, with a mean annual rate of increase of 0.243 SD (95% CI, 0.227–0.258) for the structured intervention and 0.213 SD (95% CI, 0.198–0.229) for the self-directed intervention

^#^ClinicalTrials.gov Identifier or German Clinical Trials Register (DRKS) or International Standard Randomised Controlled Trial Number (ISRCT)

The pioneering study is the FINGER (Finnish Geriatric Intervention Study to Prevent Cognitive Impairment and Disability) study, published in 2015, which successfully demonstrated the effectiveness of such a multi-component intervention on cognitive function [16]. The study examined the effect of a two-year multidomain intervention, including nutritional counselling, exercise, cognitive training and treatment of cardiovascular risk factors, in older adults with increased risk of dementia. The results showed that the multidomain intervention improved cognitive function, particularly psychomotor speed (150%), and executive function (83%), but less on complex memory function (40%) compared to the control group.

Other lifestyle-based studies from Europe, such as the French Multi-Domain Alzheimer’s Prevention Trial (MAPT [17]), the European HATICE study [18] and the Dutch Pre-Dementia Intervention by Intensive Vascular Care (pre-DIVA [19]) study, yielded less clear results but showed benefits for cognitive function in specific subgroups at increased risk of dementia. Nevertheless, the Australian BBL-CD study [20] (2020) showed that short-term lifestyle modifications could enhance cognitive performance in individuals with subjective or mild cognitive decline.

These promising, albeit still inconsistent, findings inspired the establishment of the consortium World-Wide FINGERS (WW-FINGERS network), which was launched in 2017 and now brings together more than 40 countries conducting lifestyle studies on cognitive decline and dementia [21, 22] to adapt and optimize the FINGER model across diverse settings, aiming to identify globally effective prevention strategies.

The SMARRT study [23] examined personalised risk reduction targets in 172 adults over a period of two years through health counselling and visits from nurses. The intervention resulted in an improvement in the composite cognitive score (mean treatment effect of standard deviation 0.14, 95% CI 0.03–0.25), a 74% improvement compared to the control group, which received information about reducing the risk of dementia.

As part of the German Age-Well-Study [24] published in 2024, 1,030 adults in Germany were recruited who had above-average scores on a dementia risk calculator that considered age, gender, date of birth, height and weight, serum cholesterol, systolic and diastolic blood pressure, physical activity and educational level. The intervention encompassed the optimisation of diet and medication, in addition to physical, social and cognitive activities. Although the intervention resulted in reduced LIBRA scores, the authors concluded lacking both targetedness and intensity [24].

Finally, the POINTER-US (PrOtect brAIn health Through lifestyle Interventions to Reduce risk) study recently investigated the effects of lifestyle changes, including dietary counselling, physical activity, cognitive training and self-monitoring, on the cognitive function of older adults at increased risk of cognitive decline [25]. The results showed that both structured and self-directed interventions led to improvements in global cognitive function. However, the structured intervention group showed significantly greater improvements compared to the self-directed group. The study suggests that structured multidomain lifestyle interventions may be more effective in improving cognitive function, underscoring the importance of guidance and support in promoting lifestyle changes for the benefit of cognitive health.

In this context, it should be noted that RCTs are considered the gold standard for assessing the effectiveness of interventions and the causality of risk factors, but they are often just not feasible in dementia research due to long intervention periods and complex clinical trial design settings. Therefore, causal inference methods such as quasi-experimental or ecological studies need to be used to supplement the evidence. Here, a Cochrane review showed that 23 out of 34 studies reported comparable effect estimates from RCTs and observational studies, but differences occurred in cases of high heterogeneity [26]. These findings highlight the challenges of conducting RCTs in dementia research and the importance of alternative study approaches to supplement the evidence.

Taken together, these clinical trials suggest that multidomain interventions, dietary changes, cognitive training and the treatment of cardiovascular risk factors are promising for the prevention of dementia.

### Current state on primordial, primary, secondary, tertiary and possibly also quaternary prevention in cognitive decline and dementia

**Fig. 1:**
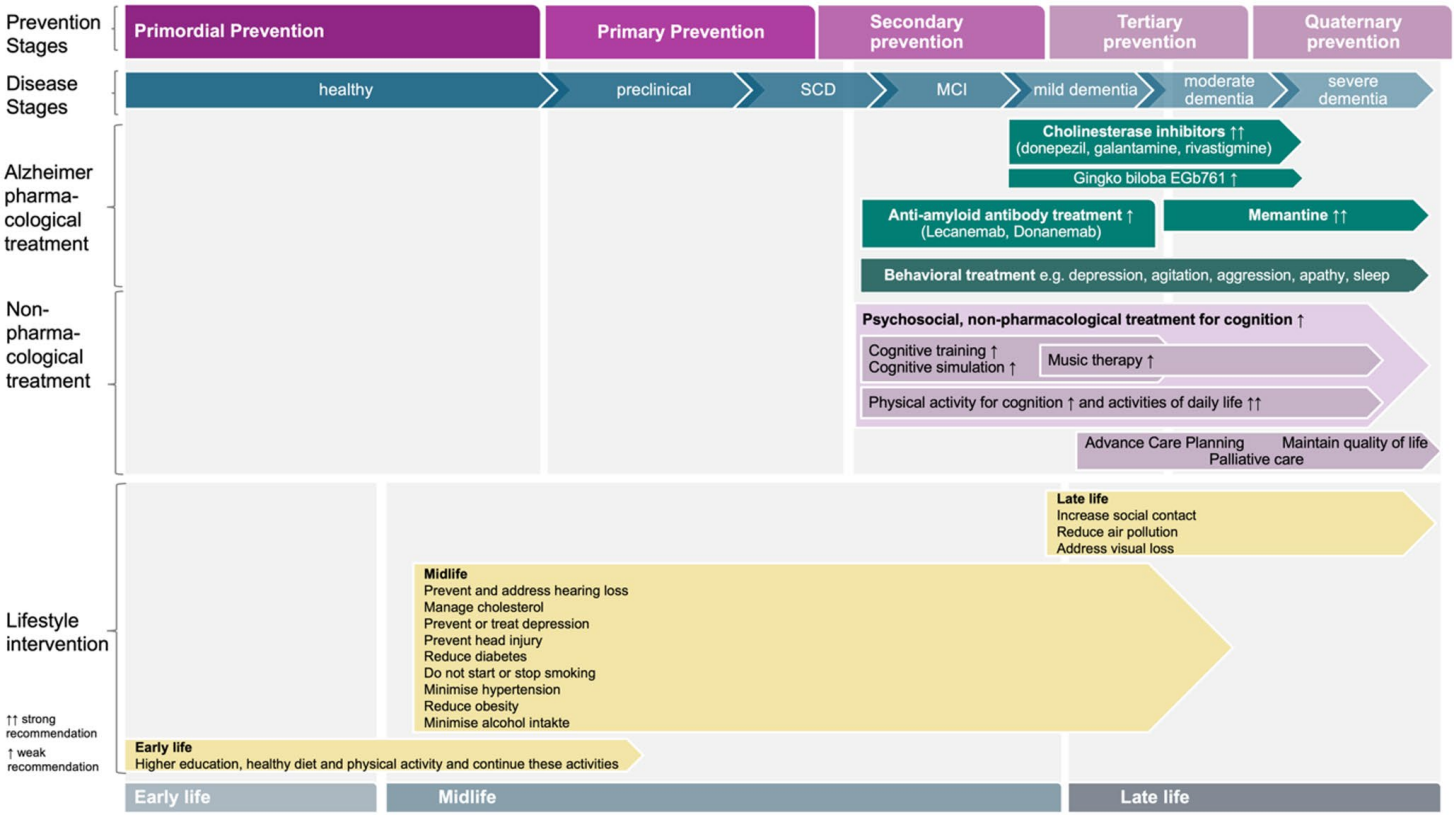
Schematic overview about timing of prevention stages and pharmacological and non-pharmacological treatment as well as lifestyle intervention in the context with Alzheimer staging. For patients with mild cognitive impairment and mild dementia due to Alzheimer’s disease, who have biomarker confirmation of Alzheimer pathology by CSF or PET, no homozygous APO4 status, receive no anticoagulants and show no distinct pathological signs on brain MR imaging monoclonal anti-amyloid treatment is now available. For patients with mild and moderate dementia due to Alzheimer’s disease acetylcholine inhibitors such as donepezil, galantamine and rivastigmine (↑↑ strong recommendation) and Gingko biloba EGb761 (↑ weak recommendation) are used for the symptomatic treatment, and the NMDA-agonist memantine for moderate and severe dementia (↑↑ strong recommendation) according to German national guidelines dementia [10]. Psychosocial, non-pharmacological treatment for cognition, in particular cognitive training and cognitive simulation as well as physical activity is recommended for secondary and tertiary prevention, and for latter as well music therapy (strong to weak recommendation, partly based on expert consensus according to German national guidelines dementia [10]). Lifestyle modification includes eliminating major risk factors such as reducing diabetes, managing cholesterol, minimising hypertension, stop smoking, reducing obesity, reducing air pollution, preventing head injury, preventing or treating depression, regular exercising (physical activity), minimising alcohol intake, higher education or continue cognitive activity, preventing and addressing hearing loss, addressing visual loss and increasing social contacts, according to Livingston et al., [32]. Abbr.: SCD, subjective cognitive decline; MCI, mild cognitive impairment

According to the natural history of the disease, preventive health measures can be grouped accordingly to target the prevention of these stages of the disease. For cognitive decline and dementia, the natural history order is as follows: normal cognition, subjective cognitive decline (SCD), mild cognitive impairment (MCI), mild dementia, moderate dementia, and finally, severe dementia (Fig. 1).

The earliest prevention modality is *primordial prevention* targeting children and youth to decrease as much risk as possible by addressing social determinants, public health policies and early life interventions (Table 2).

**Table 2:** Overview about prevention stages, target population and strategies

Type	Target population	Measures & Objectives
Primordial prevention	Children and Youth	Increasing education, healthy diet and physical activity
Primary prevention	Whole/susceptible population, in particular individuals at an elevated risk of developing cognitive decline	Identifying and managing risk factors for cognitive decline
Secondary prevention	Individuals with mild cognitive impairment	Screening for early diagnosis, cognitive and physical training, lifestyle modification, managing comorbidities, treatment with anti-amyloid antibodies
Tertiary prevention	Individuals with dementia	Cause diagnostics, pharmacologic agents such as cholinesterase inhibitors (donepezil, galantamine, rivastigmine) and memantine as well as Gingko biloba EGb761; non-pharmacological approaches include cognitive therapies, behavioral management
Quaternary prevention	Individuals with advanced dementia	Quality of life, evaluate over-medicalization, palliative and end-of-life care

*Primary prevention* is about stopping the onset of mental health conditions and is aimed at the general population. Here most relevant are the modifiable 14 risk factors for preventing or delaying dementia, which have been outlined in the 2024 report of the *Lancet* standing Commission [6]. Evidence points to the lessons ‘the earlier and longer, the better’ and ‘it is never too early or too late to reduce’ [6]. Consequently, multicomponent intervention is not only relevant for primary but also secondary and even tertiary prevention.

*Secondary prevention* targets the prodromal disease stage, with the focus thus being on early disease detection. Multicomponent interventions can be implemented at this stage, including cognitive and physical training, lifestyle modification, and the management of comorbidities. In addition, recently approved anti-amyloid antibody treatments with lecanemab and donanemab for early symptomatic AD qualify as secondary intervention strategies. Current studies are investigating whether patients who are at increased risk of developing symptoms of AD due to certain biomarkers can reduce their risk of cognitive decline with lecanemab (AHEAD 3–45 study [27]) or donanemab (TRAILBLAZER-ALZ 3 trial [28]) treatment. *Tertiary prevention* targets individuals with established disease, aiming to reduce severity and prevent complications. It focuses on managing symptoms and improving quality of life through interventions like pharmacological and non-pharmacological approaches (for more details see below) [10].

*Quaternary prevention* in dementia aims to avoid unnecessary or harmful interventions in individuals with dementia. Its main objectives are to minimize overmedicalization, preserve dignity, and maintain quality of life. Measures involve careful assessment before prescribing, avoiding futile or invasive procedures, promoting palliative care, and ensuring ethical decision-making. Wellbeing and quality of life for persons in late stage through advance care planning should be promoted [29].

### Gaps in knowledge in the field

There are several gaps in knowledge in the field. First, widespread stigma, misconceptions and low awareness of cognitive decline and dementia, including lack of knowledge about prevention and risk factors, hinder health-seeking behavior. *Knowledge of risk and protective factors for dementia is insufficient*, and more information and intervention approaches are needed in the population [30, 31]. Here, public awareness campaigns are vital to counter stigma and promote understanding, starting in kindergarten, schools, workplaces, and in public for a next generation brain health.

Second, as the Lancet Commission on Prevention of Dementia [32] pointed out, for several potential risk factors, we have so far *insufficient evidence* to include them in the recommendations. These – also potentially modifiable risk factors – comprise too little or poor quality of sleep, an unhealthy diet, infections, and mental health conditions. For example, based on epidemiology, several lines of evidence indicate that short sleep duration might be associated with a small, increased risk of dementia [33], but evidence about the characterization of short sleep is scarce, and little information exists on sleep quality or circadian rhythm disturbance, which might be the factors associated with increased risk of developing dementia rather than length of sleep. Therefore, Livingston et al. concluded that the evidence about short sleep has not yet been clarified enough to be sure of causation, so no recommendation could be made; however, sleep has already been included in the updated LIBRA2 score [34]. However, this question should certainly be addressed in future work: It has been demonstrated that sleep disturbances often occur years before the first clinical symptoms appear and are associated with the accumulation of neurotoxic proteins such as β-amyloid (Aβ) and tau [35]. There is a bidirectional relationship between AD pathophysiology and sleep. Given the link between sleep and AD pathology, future experimental work, as well as clinical proof of concept studies, will help to further elucidate this promising avenue. Another example is nutrition. A healthy diet might be an important factor in addition to the 14 factors described by Livingston et al., as it has a key role in reducing the risk of chronic diseases that affect dementia risk, and it is an important component of multidomain approaches to dementia risk reduction.

### Gaps in action in the field

Several hurdles hinder the translation of current knowledge about prevention options into actual health improvements:

*Failure of behavioral prevention*. A central challenge in prevention is the well-documented intention–behavior gap: although many individuals express a desire to adopt healthier lifestyles, only about half of these intentions translate into sustained behavioral change [36]. This gap is strongly shaped by structural and environmental conditions, such as limited financial resources, lack of safe spaces for physical activity, and residence in food deserts or other obesogenic environments, that restrict people’s ability to act on their intentions. Digital offerings (e.g., fitness apps and wearables) have not been shown to close this gap. Their evidence base remains limited, and they primarily reach individuals who already have the resources, health literacy, and motivation to engage in preventive behaviors. They rarely address the needs of those facing the greatest structural barriers, who may not proactively seek help and who represent a substantial proportion of hospital patients [37]. These tools are also often disconnected from local services that could support low-threshold access to preventive resources. These structural factors contribute to the persistently low reach of behavior-based prevention approaches among groups most in need. Individuals with low socioeconomic status, multimorbidity, or multiple behavior-related risk factors are disproportionately affected by adverse Social Determinants of Health (SDoH) and are often the least likely to be reached by conventional strategies [38]. Ensuring that prevention efforts effectively engage these groups requires approaches that directly address structural barriers, for example, integrating preventive services into community settings, strengthening links between healthcare providers and local social support systems, tailoring interventions to local resources, and reducing financial and geographic obstacles to healthy choices. Such measures are essential for improving population health.

*Lack of structural prevention*. Structural or environmental prevention strategies focus on creating supportive environments that reduce risk factors and promote brain health. These may incorporate a physical environment, social engagement, health promotion, access to care, and a stimulating environment. Another important factor is the fragmentation of approaches and services: prevention approaches are offered separately from care and focus mainly on individual disease entities, patient or population groups, and technologies. This fragmented, specialist clinical approach means that opportunities for synergies and cost savings in the form of integrated, preventive care are being missed. Public health approaches also tend to focus on individual lifestyle factors (e.g., tobacco use) [39].

Last but not least, the *lack of political support* must be mentioned. This may also be due to the difficulty of quantifying the extent to which behavior-based prevention approaches can reduce costs for society. Behavior-based prevention approaches that are not targeted and tailored to the individual are less effective and therefore not cost-efficient. For such interventions, the return on prevention, i.e. the monetary return on investment in health, far exceeds the current rate of return on capital; for every euro invested, approximately two euros could be saved through the prevention of illness [40].

## How to fill these gaps and what are the next steps

Several next steps can be identified and need to be considered.

First, *making prevention of cognitive decline and dementia a priority*. Here, several lines of action are ongoing. A European task force recently developed and made public the concept and protocols for setting up innovative health services for brain health, with the aim of providing secondary prevention for dementia and cognitive decline [41]. They have identified the four pillars: (I) assessment of the risk of developing cognitive decline/dementia, (II) specific techniques and tools for the communication of that risk, (III) personalized risk reduction programs for those persons at higher risk and (IV) offering cognitive enhancement interventions [41]. However, implementation into clinical practice remains difficult.

Second, *promoting brain health throughout the lifespan through coordinated intersectoral action targeting key risk factors*. Evidence suggests that a multidomain lifestyle approach, targeting several risk factors simultaneously, is the most effective strategy for preventing or delaying cognitive decline (Fig. 1) [42, 43]. Based on the WHO recommendations for risk reduction of cognitive decline and dementia, the strength of the recommendation was strong for physical activity for adults with normal cognition, interventions for tobacco cessation, a healthy balanced diet, as well as management of hypertension and diabetes [44]. The level of strength of the recommendation was conditional for interventions aimed at reducing or ceasing hazardous and harmful drinking, cognitive interventions, weight and dyslipidemia management [44]. In humans with modifiable risk factors, the pharmacological treatment of some of these factors may delay the onset of cognitive decline and dementia. This has been shown for treating hypercholesterinemia with statins [45], treating diabetes [46] and adipositas with GLP-1 receptor agonists [47] or arterial hypertension with antihypertensives [48]. However, so far, for primordial and primary prevention, no specific medication has been conclusively proven to prevent or significantly delay the onset of cognitive decline in otherwise healthy individuals.

Third, *improving access to early detection*,* diagnosis and treatment for patients with cognitive decline and dementia*. Although there are no exact data, it is estimated that more that 50% of patients with dementia receive no diagnosis. It is recommended that patients with mild cognitive impairment and dementia are offered diagnostics to identify the cause of the syndrome, so specific treatment and dementia care management can be initiated (Fig. 1) [10]. For pharmacological treatment, the anti-amyloid monoclonal antibodies Lecanemab and Donanemab are the first approved disease-targeted therapies (DTTs) for AD, which have shown efficacy in early AD [49, 50]. However, the eligibility window for patients is small due to several criteria, the costs are high and side effects need to be monitored on regular basis. For tertiary prevention acetylcholine inhibitors such as donepezil, galantamine and rivastigmine, as well as recently Gingko biloba EGb76 are used for the symptomatic treatment of mild to moderate dementia and the NMDA-agonist memantine for moderate and severe dementia [10]. In case of depression, agitation, aggression, apathy, or sleep disturbance, behavioral symptomatic treatment should be provided. Next to the pharmacological approach, the second pillar is non-pharmacological treatment. Here, psychological therapy such as cognitive training and cognitive stimulation as well as physical activity is recommended strongly for improvement of activities of daily life and with less evidence for cognition and in more advanced staged patients music therapy [10]. Advanced care planning should be offered early on, followed by palliative care and maintaining quality of life, especially for quarternary prevention.

Finally, *investment in and encouraging research* is critical for informing strategies to prevent, accurately diagnose, and effectively treat and manage dementia, by means of for example clinical studies and trials as well as registries.

In sum, progress towards prevention and accelerate action, key operational challenges include making cognitive decline and dementia a priority, fostering brain health across all ages through coordinated efforts that address major risk factors, increasing access to diagnosis and treatment for individuals with cognitive decline and dementia, and promoting research initiatives, including the creation of registries. To overcome roadblocks, intersectional collaborations are paramount to mitigating preventable cognitive decline and dementia.

### Mission statement

The global burden of neurological diseases, increasing dramatically due to demographics, changes in lifestyle and ecotoxic influences, has a strong impact on healthcare, society, and the economy. The German Neurological Society focuses on the prevention of neurological diseases at every stage of life with strategies that advance awareness, education, individualized treatment and public health.

## Data Availability

Not applicable.

## Abbreviations

ANU-ADRI: Australian National University-Alzheimer’s disease risk index
ATE: Average treatment effects
MCI: Mild cognitive impairment
NTB: Neuropsychological test battery
SCI: Subjective cognitive impairment
SDMT: Symbol digit modalities test
